# Ivabradine for Chemotherapy-Related Cardiac Dysfunction in Breast Cancer

**DOI:** 10.7759/cureus.18731

**Published:** 2021-10-13

**Authors:** Yuko Harada, Kyosuke Shimada, Yukino Kubota, Tatsuji Yoshimoto

**Affiliations:** 1 Cardiology, Kawasaki Municipal Ida Hospital, Kawasaki, JPN; 2 Breast Surgery, Kawasaki Municipal Ida Hospital, Kawasaki, JPN; 3 Palliative Medicine, Kawasaki Municipal Ida Hospital, Kawasaki, JPN

**Keywords:** global systolic longitudinal myocardial strain (gls), heart failure with reduced ejection fraction (hfref), global systolic longitudinal myocardial strain, chemotherapy-related cardiac dysfunction (ctrcd), ivabradine, anthracycline

## Abstract

A 55-year-old woman with stage IV breast cancer was diagnosed with heart failure. Her left ventricular ejection fraction (LVEF) had decreased to 37.2%. Chemotherapy-related cardiac dysfunction (CTRCD) was suspected, and standard treatment for heart failure was initiated. After five months, her LVEF remained below 50% since she could not tolerate beta-blockers. Ivabradine was introduced, which remarkably improved her LVEF to 72.6% in only three months. Her myocardium was not dilated, which may be the reason that ivabradine was effective. Ivabradine has shown to be safe and effective in the treatment of CTRCD, and improved activities of daily living of an advanced-stage cancer patient.

## Introduction

Breast cancer is the most common cancer among women in Japan [1]. The five-year survival rate is 92.3%, and the first cause of death among female survivors of breast cancer is cardiovascular disease [1,2]. Advances in chemotherapy have reduced the mortality from breast cancer; however, the increase in survival rates has allowed observing the cardiotoxic effects of chemotherapy, which is called chemotherapy-related cardiac dysfunction (CTRCD) [3].

Here we report a case of heart failure with reduced ejection fraction (HFrEF) occurring during chemotherapy for breast cancer. HFrEF is defined as heart failure with a left ventricular ejection fraction (LVEF) less than 40% [4]. Since the patient did not tolerate standard medication for HFrEF, she was prescribed ivabradine, which greatly improved her cardiac function.

## Case presentation

A 55-year-old woman was diagnosed with breast cancer (stage IIIA, T3N1M0) six years ago. Prior to surgery, she was treated with tamoxifen, 5-fluorouracil (500mg/m^2^), epirubicin (100mg/m^2)^, cyclophosphamide (500mg/m^2^), and paclitaxel. After surgery, she was treated with tamoxifen for two years and subsequently treated with letrozole for another two years; however, the cancer relapsed. She was initiated on new chemotherapy; however, she had to change anti-cancer drugs due to the adverse side effects of fulvestrant, palbociclib, abemaciclib, paclitaxel, bevacizumab, and medroxyprogesterone acetate, consecutively. Even with chemotherapy, the cancer progressed and metastasized to the bones and liver. She developed dyspnea and systemic edema, and her body weight increased by 10kg in three months.

**Figure 1:**
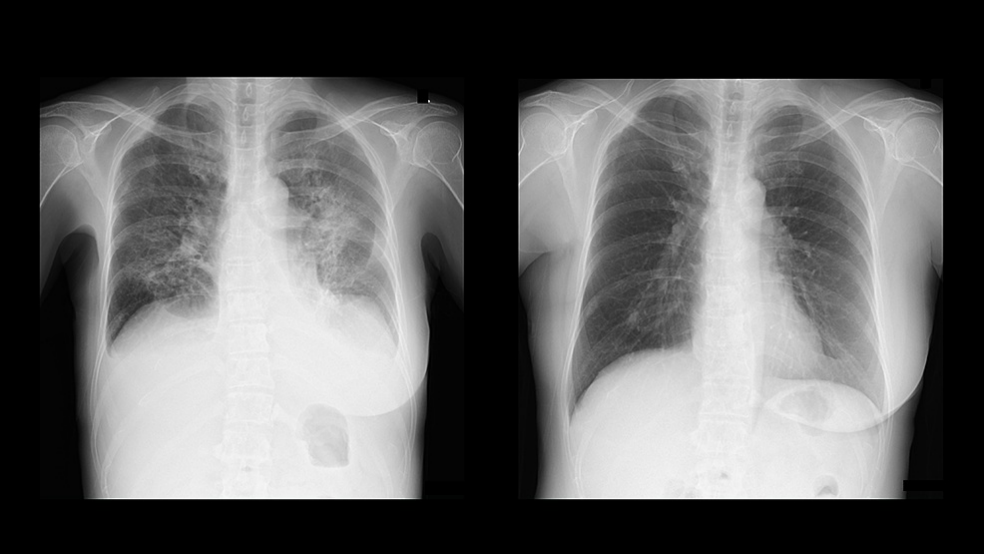
Chest X-ray before and after treatment. Left: chest X-ray on admission revealed remarkable pulmonary congestion. Right: chest X-ray after eight months.

On admission, her blood pressure was 134/94mmHg, heart rate was 112 beats/minute, and oxygen saturation was 92% (room air). She presented with remarkable edema in both legs. Chest X-ray revealed remarkable pulmonary congestion (Figure 1). Creatinine phosphokinase was only 132 IU/L; however, brain natriuretic peptide (BNP) was elevated to 1,747.4 pg/mL. Electrocardiogram revealed sinus tachycardia without ST level change. Echocardiogram revealed diffuse hypo-kinetic heart with reduced LVEF of 37.2%, mild mitral valve regurgitation, and global systolic longitudinal myocardial strain (GLS) of 5.4%.

**Figure 2:**
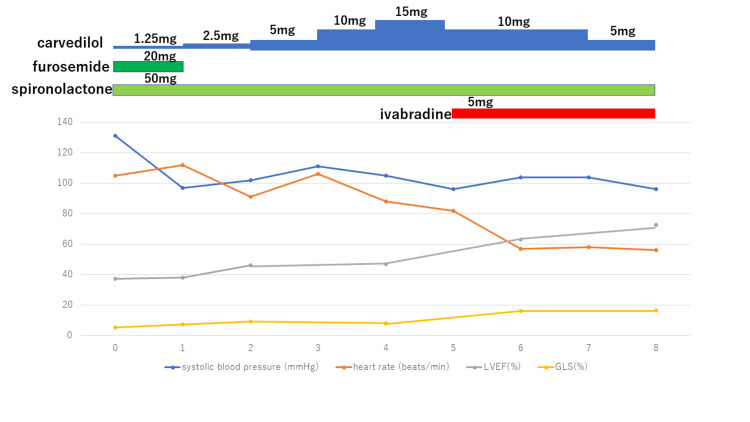
Clinical course and treatment. Clinical course of eight months since admission. Left ventricular ejection fraction and global systolic longitudinal myocardial strain remarkably improved after initiating ivabradine.

The patient was diagnosed with HFrEF and was treated with diuretics, mineralocorticoid receptor antagonist (MRA), and beta-blocker. Diuretics was used to treat systemic edema and pleural effusion; however, her systolic blood pressure dropped to 90 mmHg. Therefore, it was difficult to add angiotensin-converting enzyme inhibitor (ACEI) to the minimal dose of beta-blocker. MRA was continued to treat edema and pleural effusion. Her symptoms of dyspnea and edema were resolved, and she was discharged from the hospital in three weeks. However, her LVEF remained below 40%. Beta-blocker carvedilol was initiated to improve cardiac function, but it proved difficult to increase dosage due to her low blood pressure of 85-100mmHg. We tried to discontinue MRA, but her edema relapsed. At five months, her LVEF remained at approximately 40%, and she was not able to walk for more than 10 minutes. Thus, rather than increasing beta-blockers, ivabradine was initiated instead. As a result, her LVEF was normalized to 72.6%, BNP decreased to 34.6 pg/mL, and her exercise endurance was improved in only three months (Figure 2).

The echocardiogram also revealed remarkable improvement in LVEF and GLS (Table 1).

**Table 1:** Parameters of echocardiogram during treatment. LVEF, GLS, and MR improved after initiating ivabradine at five months. Note that the patient had a small heart. LVEDd, left ventricular end-diastolic diameter; LVESd, left ventricular end-systolic diameter; LVEF, left ventricular ejection fraction; GLS, global systolic longitudinal myocardial strain; MR, mitral regurgitation

	Time (months)					
	0	1	2	4	6	8
LVEDd (mm)	42	41	32	31	35	39
LVESd (mm)	36	31	25	25	22	25
LVEF (%)	37.2	38	46.1	47	63.2	72.6
GLS (%)	5.4	7.4	9.2	8.2	16.2	16.4
MR	++	++	+	+	+	+

## Discussion

The patient did not have any risk factors for ischemic heart disease nor a family history of cardiac disease. Her sequential echocardiogram findings were normal until three months prior to admission. Therefore, primary cardiomyopathy and ischemic heart disease were excluded from the differential diagnosis. The patient was subsequently diagnosed with HFrEF due to secondary cardiomyopathy. Drug-induced cardiomyopathy was most plausible; however, well-known cardiotoxic agents such as anthracycline or cyclophosphamide were used years ago, and each dose was small. Anthracycline-induced cardiomyopathy is known to be dose dependent and increases with cumulative dose over 400mg/m^2^ [3]. However, the patient used anthracycline only 100mg/m^2^ in total. The other anti-cancer drugs may have caused cardiomyopathy together or may have interacted with each other, but the doses of each drug were small.

CTRCD is difficult to prevent and predict. Even though the patient was carefully monitored, and echocardiogram had shown normal findings until recently, the patient developed CTRCD. Prevention and treatment protocols for CTRCD are not yet established. CTRCD is defined as a decrease in the LVEF of >10 percentage points to a value <53% (normal reference value for two-dimensional echocardiography) [5]. Another parameter, GLS, is considered to be the gold standard for detecting early left ventricular (LV) dysfunction in patients treated with cardiotoxic chemotherapy; a relative percentage reduction of GLS of 15% from baseline is considered abnormal and a marker of early LV subclinical dysfunction [3,6].

There is currently no specific treatment for CTRCD. Therefore, it is treated as chronic heart failure due to other causes. Standard medications for HFrEF are beta-blocker and ACEI or angiotensin receptor blocker (ARB) [7]. However, some patients with HFrEF are not able to tolerate these medications due to low blood pressure.

Ivabradine is a new therapeutic agent that selectively inhibits Iƒ current in the sinoatrial node, providing heart rate reduction [7]. It is recommended in the U.S. guidelines for the treatment of HFrEF to initiate and up-titrate beta-blockers to target doses before consideration of ivabradine initiation [7]. In the updated Japanese guideline, ivabradine is also recommended as an additional drug in treating HFrEF with sinus rhythm and heart rate of 75 beats/minutes or higher [4].

An advantage of ivabradine is its safety. It can be used for chronic kidney disease, chronic liver disease, and/or chronic lung disease. Ivabradine has drawn attention as an optimal medication for HFrEF with hypotension and other organ failures to improve ejection fraction (EF) with minimum side effects. Ivabradine is also a promising medication for CTRCD, which usually presents with HFrEF and/or other organ failures.

There are only a few reports on ivabradine use for CTRCD [8-10]. Nakano et al. reported a case of CTRCD by malignant lymphoma, which showed improvement in HFrEF with treatment including ivabradine, but the follow-up period was 30 days [8]. Sarocchi et al. reported a cohort study of 30 patients with CTRCD receiving ivabradine on top of the maximal tolerated dose of ACEI/ARB and beta-blockers, which revealed an increase in LVEF from 45.1% to 53.2% after a mean follow-up of 6.5 months [9]. None of the previous studies reported any adverse effect of ivabradine. The present case has demonstrated significant recovery of EF from 37.2% to 72.6% and of GLS from 5.4% to 16.4% in only three months and furthermore without any adverse effects. As indicated in previous reports, ivabradine has therefore shown to be effective within just a few months.

It is unclear why ivabradine was effective for CTRCD. In the present case, left ventricular end-diastolic diameter (LVEDd) was only 42 mm on admission, which did not change so much from six years ago when LVEDd was 40mm. Therefore, the cardiac muscle may not have been entirely damaged. Ivabradine may have successfully restored the optimal heart rate to recondition the cardiac muscle. Further studies are needed to reconfirm the reasons why ivabradine is effective for CTRCD.

Ivabradine is optimal for treating CTRCD. The purpose of treating CTRCD is to improve both prognosis and activities of daily living (ADL). Furthermore, ivabradine takes effect within a very short period of time. It is suitable especially for patients with hypotension and/or other organ failures, as it has fewer side effects than other medications.

The follow-up period of our study was less than one year, but the patient was able to enjoy a normal life, which is important for advanced-stage cancer patients.

## Conclusions

Ivabradine successfully treated CTRCD, which occurred in a middle-aged cancer patient who could not tolerate beta-blockers. Ivabradine was shown optimal for treating CTRCD as well as improving symptoms and ADL. Further clinical studies are anticipated to determine conditions for initiating such medication.
